# Temporal complementarity in lower-limb neuromuscular control and kinetic asymmetries: implications for elite speed-climbing performance

**DOI:** 10.3389/fphys.2026.1916621

**Published:** 2026-09-03

**Authors:** Fuxin Hong, Jungwoon Seo, Zhengxiong Zhu, Ming Li, Daehee Kim

**Affiliations:** 1 College of Education and Sports Sciences, Yangtze University, Jingzhou, China; 2 Department of Physical Education, Pukyong National University, Busan, Republic of Korea; 3 Department of Physical Education, Dongguk University, Seoul, Republic of Korea; 4 Postdoctoral Research Station, Chengdu Sport University, Chengdu, China

**Keywords:** climbing time, co-contraction index, electromyography, ground reaction force, statistical parametric mapping

## Abstract

**Purpose:**

This study applied statistical parametric mapping (SPM) to examine temporal differences in bilateral lower limb neuromuscular control and kinetics during unilateral countermovement jumps (CMJ) in speed climbers, and to explore their relationship with sport-specific performance.

**Methods:**

Fourteen elite male climbers completed unilateral CMJs and a standard 15 m speed climb. Surface electromyography and vertical ground reaction force (vGRF) were synchronously recorded. Root mean square amplitudes, knee/ankle co-contraction indices (CCI), and time-varying vGRF curves were computed, alongside asymmetry indices. SPM assessed bilateral temporal differences across the CMJ cycle, while Pearson correlation evaluated links between asymmetry and climbing time.

**Results:**

SPM results showed significantly higher activation of rectus femoris, biceps femoris, and tibialis anterior, as well as greater knee and ankle CCI in the left limb at specific phases (p < 0.05). vGRF exhibited phase-dependent asymmetry: the left leg produced greater force during rapid loading (30–32%), whereas the right leg showed higher output during the peak phase (90–94%). Climbing time was positively correlated with vGRF asymmetry (r = 0.61, p = 0.028), but not with CCI asymmetry (p > 0.05).

**Conclusion:**

Results indicate clear functional differentiation. The left limb showed higher activation and co-contraction during braking, propulsion, and peak phases, primarily for stabilization. The right limb generated higher force during propulsion and peak phases, serving an explosive role. Notably, only vGRF asymmetry, and not neuromuscular asymmetry, was associated with performance. These findings suggest that training may prioritize balancing macroscopic kinetic output (i.e., reducing vGRF asymmetry) rather than pursuing inter-limb neuromuscular symmetry.

## 1 Introduction

Speed climbing requires athletes to complete a standardized 15 m route in the shortest possible time. The fundamental objective is to achieve high-speed vertical displacement of the body, which places substantial demands on explosive strength and speed capacity (Fuss et al., 2020; Li et al., 2026). Existing studies consistently indicate that strength and speed are critical physical determinants of climbing performance (Krawczyk et al., 2020; Ehiogu et al., 2023; Zhang et al., 2025). Among these, lower limb capacity is particularly important and is considered a key factor in determining climbing efficiency (Krawczyk and Ozimek, 2014; Ehiogu et al., 2023). For example, Ehiogu et al. (2023) noted in their review of strength characteristics in speed climbers that vertical jump height and lower limb maximal strength are significantly higher than in athletes from other climbing disciplines. They further emphasized that the functional contribution of the lower limbs in speed climbing is approximately 1.8 times that of the upper limbs. As a result, current research on speed climbing often focuses on lower limb strength assessment. Winkler et al. (2023) included both unilateral and bilateral countermovement jumps (CMJ) as part of a broader lower-limb power test battery in speed climbers. In their preliminary studies, this test battery demonstrated high reliability (all intraclass correlation coefficients ICC > 0.86) and validity (correlation coefficients r > 0.52). Using factor analysis, they extracted a lower-limb power factor, supporting the practical value of CMJ-based measures for predicting speed-climbing performance.

In addition to its reliance on strength and speed, speed climbing is characterized by a highly non-cyclical and rhythmically precise movement structure. Such non-cyclical movements often display clear bilateral asymmetry, leading to differences in load magnitude and application time across joints (Hartley et al., 2023). Long-term training may induce functionally distinct adaptations between limbs. For example, Zhang et al. (2025) reported bilateral asymmetries in the rate of force development in speed climbers, with the left lower limb outperforming the right. Similarly, Guo et al. (2019) observed a general tendency toward greater fatigue-related decreases in sEMG median frequency in the left than the right limbs during a 15-m speed climb, although this pattern was most evident in the upper-limb muscles.

Limb asymmetry is widely regarded as an important factor associated with athletic performance. Studies have shown that when the lower limb strength asymmetry index exceeds 10%, unilateral CMJ jump height decreases significantly (Bell et al., 2014). Recently, Zhang et al. (2025) further confirmed that asymmetries in peak force and rate of force development are both significantly positively correlated with speed climbing performance. Although existing research has consistently reported the presence of inter-limb asymmetry in climbers and its potential negative impact on performance, most studies focus on macroscopic mechanical or temporal parameters. These often rely on discrete indicators such as means or peak values to summarize complex movement processes. For example, previous studies have investigated bilateral arm recovery time (Giles et al., 2017), peak finger flexor strength (Hartley et al., 2023), and bilateral lower limb peak force asymmetry (Zhang et al., 2025), but have paid less attention to the key time-varying features and fluctuations during movement execution, as well as the underlying control mechanisms. In particular, the neuromuscular regulatory mechanisms behind asymmetry—especially muscle co-contraction, a key indicator of joint dynamic stability—remain underexplored. The co-contraction index (CCI) reflects the central nervous system’s strategy for actively regulating joint stiffness (Baratta et al., 1988; Latash, 2018) and directly affects energy transfer efficiency and disturbance resistance in high-speed, non-cyclical movements (Butler et al., 2003; Cormie et al., 2011). Unlike EMG amplitude, which reflects the activation of individual muscles, or vGRF, which captures the net output of the whole limb, CCI characterizes how agonist and antagonist muscles are coordinated around a joint, and the knee and ankle were therefore examined as the principal joints governing force generation and postural control during high-speed push-off. Neglecting asymmetries at this neuromuscular control level may limit a deeper understanding of the mechanisms constraining performance in speed climbing. Therefore, a systematic investigation of limb asymmetry from the perspective of neuromuscular regulation, particularly muscle co-contraction, holds significant theoretical and practical value for revealing the underlying mechanisms of performance in speed climbing.

This study aimed to apply statistical parametric mapping (SPM) to analyze detailed temporal differences between left and right limbs throughout the full cycle of unilateral jumping. The focus was on major muscle activation, co-contraction indices (CCI) of the knee and ankle joints, and vertical ground reaction force (vGRF). It further explored the asymmetry characteristics of bilateral knee and ankle joint CCI and vGRF, and their relationships with speed climbing performance (climbing time). The goal was to reveal how speed climbers maintain balance in macroscopic kinetic output during high-speed, non-cyclical movements through fine-scale neuromuscular compensatory strategies, and to assess the potential impact of such mechanisms on sport-specific performance. Based on existing research, this study hypothesized that speed climbers may exhibit temporal differences in neuromuscular control and kinetic output characteristics between limbs during key movement phases, with varying degrees of asymmetry at the ankle and knee joint levels. In addition, macroscopic kinetic asymmetry may show a significant correlation with speed climbing performance, while the relationship between neuromuscular control asymmetry and performance remains to be further validated.

## 2 Methods

### 2.1 Experimental design

This study adopted a cross-sectional experimental design. All participants completed the entire testing protocol within the same time period to control for potential circadian rhythm effects. The experimental procedure began with a standardized 25-minute warm-up, including 5 minutes of low-intensity jogging, 14 minutes of dynamic stretching, and two submaximal 15 m speed climbs aimed at activating the lower limb muscles and integrating sport-specific movements. A 3-minute active recovery period was provided between the two climbs. Following the warm-up, participants rested passively for 5 minutes and then completed the single-leg countermovement jump (CMJ) and 15 m speed-climbing tests. The order of the two testing tasks was determined using a computer-generated randomization sequence. Within the single-leg CMJ test, the order of limb testing was also randomized and counterbalanced: eight participants performed the left-leg trials first, whereas seven participants performed the right-leg trials first. This procedure was used to minimize potential learning, order, and fatigue effects. A 3-minute passive recovery period was provided between consecutive jump trials, and a 5-minute passive recovery period was provided before and after the speed-climbing test. To ensure data quality and control potential confounding factors, participants were instructed to avoid strenuous exercise and alcohol consumption for 48 hours before testing, refrain from consuming caffeinated beverages for 8 hours before testing, and abstain from food intake for 2 hours before testing.

### 2.2 Participants

A total of 14 male speed climbers participated in this study. Their basic characteristics were as follows: age 20.13 ± 0.92 years, height 171.3 ± 6.27 cm, body mass 63.6 ± 5.79 kg, and training experience 5.5 ± 1.86 years. All participants were high-level competitive athletes with experience in the China University Rock Climbing Championships. In the most recent competition, they achieved outstanding results, including one first-place, three second-place finishers, and the remaining athletes ranked within the top six. Their personal best times in the 15 m speed climb ranged from 5.94 to 10.53 seconds. Inclusion criteria required that participants had no neuromuscular disorders or relevant medical history and had been consistently engaged in systematic sport-specific training (5–6 sessions per week, each lasting 3–3.5 hours). Prior to the experiment, all procedures and potential risks were fully explained to the participants. Written informed consent was obtained from each participant after understanding the provided information. The study protocol was approved by the university ethics committee (approval number: 202596), and all procedures strictly complied with the ethical principles outlined in the Declaration of Helsinki for research involving human subjects.

### 2.3 Preparation for testing

A synchronized acquisition system was used to simultaneously record kinetic and electromyographic data. Vertical ground reaction force was collected using an AMTI force platform (Model: BP600900; Advanced Mechanical Technology, Inc., Watertown, MA, USA) at a sampling frequency of 2000 Hz. Surface electromyography (sEMG) was used to record bilateral muscle activity of the rectus femoris (RF), biceps femoris (BF), tibialis anterior (TA), and medial gastrocnemius (MG). The system employed was the Noraxon ULTIUM-ESP wireless sensor system (Noraxon USA, Inc.; dimensions/weight: 37×24.5×16.5 mm, 14 g). The sEMG system was configured with a 2000 Hz sampling rate and a ±24 mV input range. It also featured high common-mode rejection ratio (CMRR > 100 dB) and high input impedance (>1000 MΩ) differential amplifiers to ensure signal accuracy and stability. Prior to electrode placement, skin preparation involved shaving and alcohol cleaning to reduce skin impedance. Bipolar Ag/AgCl surface electrodes (Ambu^®^ BlueSensor N; size: 30×22 mm) were applied with an inter-electrode distance of 1 cm, aligned along the muscle fiber direction. To minimize motion artifacts, the sensors were secured with athletic tape (Besomi et al., 2024). All data were collected using Noraxon MR 3.20.68 software to ensure temporal synchronization and consistent processing of kinetic and electromyographic signals.

### 2.4 SCMJ test

Single-leg countermovement jumps (SCMJ) were used to assess bilateral lower-limb function, as this task isolates unilateral force production and neuromuscular control and is a well-established, climbing-relevant indicator of lower-limb capacity in speed climbers (Krawczyk et al., 2020; Winkler et al., 2023; Zhang et al., 2025). This study adopted the standardized testing protocol proposed by Yanci and Camara (2016). Each participant completed a total of six valid single-leg CMJ, including three trials per leg in randomized order. Prior to formal testing, participants performed one successful single-leg CMJ using their dominant leg to determine their preferred squat depth during the eccentric phase. This depth was recorded and used as a standard reference for all subsequent trials. During testing, participants placed their hands on their hips and stood on one leg. They then performed a quick self-selected depth squat followed by a maximal vertical jump, landing with both feet for cushioning. A 60-second passive rest was provided between jumps to ensure adequate recovery. Trials were excluded and repeated if the movement was discontinuous or if maximal effort was not exerted.

### 2.5 Speed climbing test

The speed climbing test was conducted on a 15-meter standard speed route certified by the International Federation of Sport Climbing. The wall had a 5° overhang and was equipped with standardized hold configurations. Climbing time was accurately recorded using an electronic timing system with optical sensors integrated at the start and finish points. All participants adopted a standardized starting posture: both hands gripping the designated start holds, the left forefoot contacting the timing pad, the right forefoot positioned on the start hold with the heel raised. The torso was leaned forward at approximately 45° to 60°, and the hips were lowered to optimize storage of elastic potential energy in the lower limbs. The test began upon confirmation of a stable starting posture, followed by a verbal cue. Participants then initiated an explosive start and completed the climb at maximal speed, stopping the timer by touching the top sensor. Each participant performed three maximal-effort climbing attempts, with a 5-minute rest between trials to ensure full recovery. The fastest of the three trials was selected for statistical analysis. To ensure safety and data integrity, all tests were conducted using standard safety equipment and were supervised by certified professionals.

### 2.6 Data processing

The vGRF and sEMG data collected during the single-leg CMJ test were saved in C3D format and processed using Visual3D Setup ×64 v2024.9.1 software. vGRF data were first smoothed using a fourth-order zero-lag Butterworth low-pass filter with a cutoff frequency of 20 Hz. Based on this, the movement phase was defined as follows: the onset of motion was identified when the vGRF dropped more than 20 N below the static body weight, and the end of motion was when the vGRF dropped below 20 N (McMahon et al., 2018). The extracted force-time data were processed in two ways: (1) mean values for each phase were calculated for asymmetry index analysis, and (2) the data were normalized to 101 points for SPM analysis (Pataky, 2010). sEMG signals were synchronously extracted during the jump phase. They were pre-processed using a fourth-order Butterworth band-pass filter with a frequency range of 20–500 Hz. Root mean square (RMS) values were then calculated; these amplitudes were expressed in arbitrary units and were not normalized to MVC, as all comparisons were made within participants between the left and right limbs using bilaterally symmetrical electrode placement. These RMS values were used for two types of analysis: (1) normalization to 101 points for SPM-based statistical analysis, and (2) calculation of co-contraction indices (CCI) for muscle pairs (RF-BF, TA-MG), denoted as CCI_RF-BF_ and CCI_TA-MG_. The resulting CCI curves were also normalized to 101 data points for statistical analysis. Total CCI values were used to compute CCI asymmetry.

The co-contraction index was calculated according to the method proposed by Larsen et al. (2008):


$$\text{CCI}=\frac{2\times \min({\text{RMS}}_{1},\;{\text{RMS}}_{2})}{{\text{RMS}}_{1}+{\text{RMS}}_{2}}\times 100\%$$

The asymmetry index was calculated following the method described by Schmidt et al. (2016):


$$\text{Asymmetry Index}=200\times \frac{\left|\text{Left}-\text{Right}\right|}{\text{Left}+\text{Right}}\%$$

The asymmetry index was computed for each participant individually and then averaged across the sample. In addition, to ensure the reliability of the test data, the force data from the three trials were subjected to a consistency check, using a coefficient of variation below 10% as the criterion (Zhang et al., 2025). If the three trials met this requirement, their average was used for subsequent analysis; otherwise, the data for the same condition were re-collected within the same time period.

### 2.7 Statistics analysis

Statistical analyses were conducted using customized Python 3.13 scripts running in PyCharm 2024.1 (Professional Edition; JetBrains, Prague, Czech Republic) (Pataky, 2012). All data are presented as mean ± standard deviation and were tested for normality using the Shapiro–Wilk test (p > 0.05) prior to analysis. SPM was used to compare the RMS of bilateral major muscles, CCI_RF-BF_, CCI_TA-MG_, and vGRF across the entire movement cycle. Specifically, SPM paired samples t-tests were performed to calculate the statistical trajectory SPM{t}. To address the multiple comparisons problem and control the Type I error rate, Random Field Theory was applied to determine the critical threshold at a significance level of α = 0.05. Time periods where the SPM{t} trajectory exceeded this threshold were identified as “suprathreshold clusters.” Each cluster was then assigned a p-value to assess statistical significance. A time period was considered to show a significant bilateral difference if p < 0.05. For each suprathreshold cluster, the peak effect size (Cohen’s d) was calculated to quantify the magnitude of the bilateral difference. Additionally, Pearson correlation analysis was used to evaluate the relationships between climbing performance and the asymmetry indices of vGRF (ASI_vGRF_), CCI_RF-BF_ (ASI_RF-BF_), and CCI_TA-MG_ (ASI_TA-MG_). The interpretation of correlation coefficients followed Hopkins’ scale: trivial (0–0.1), small (0.1–0.3), moderate (0.3–0.5), large (0.5–0.7), and very large (0.7–0.9). A statistically significant correlation was defined as r ≥ 0.25 and p < 0.05.

## 3 Results

**Figure 1:**
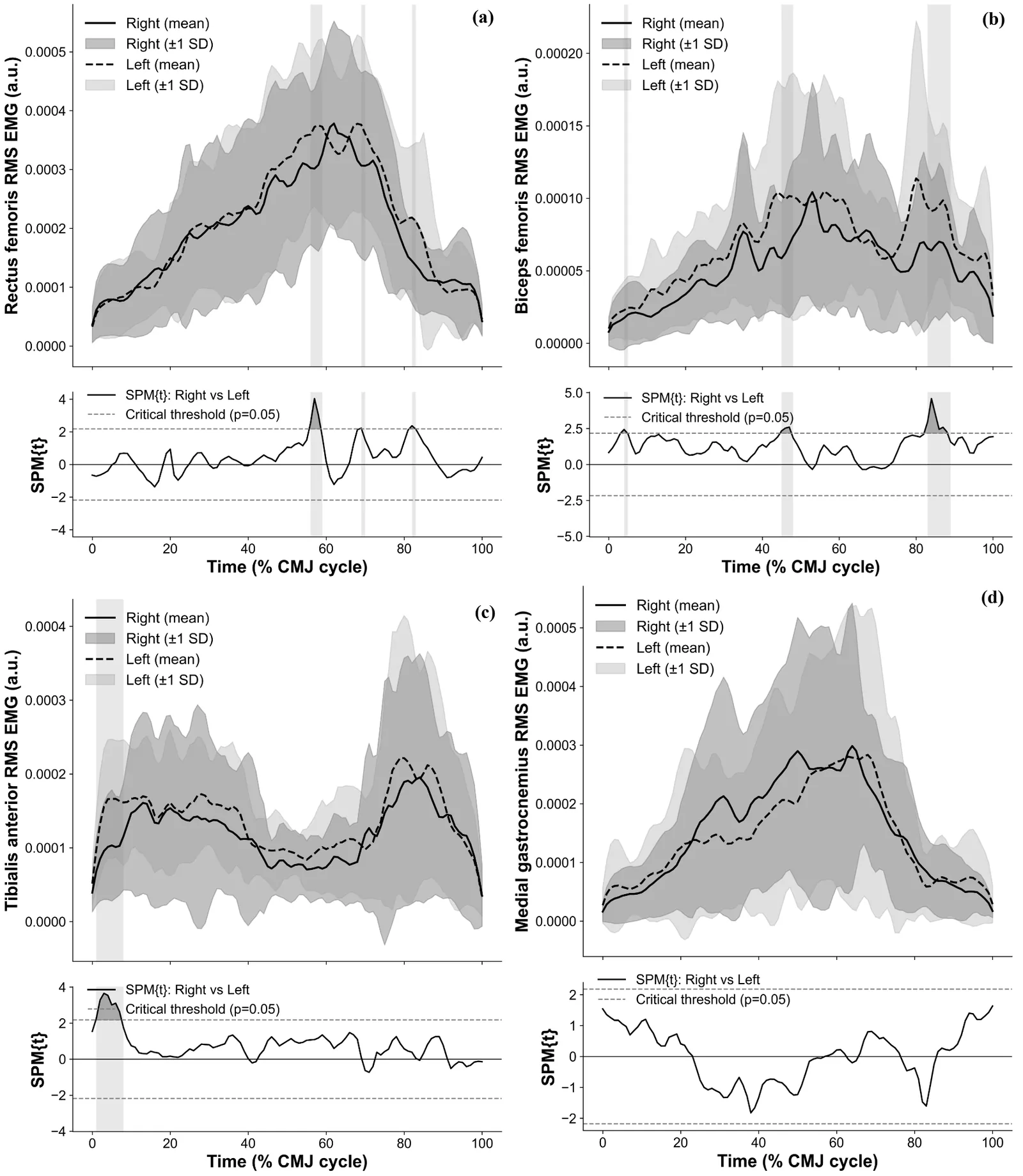
Statistical parametric mapping (SPM) analysis of EMG RMS amplitude for major lower limb muscles during the single-leg countermovement jump (CMJ). Panels represent **(A)** Rectus Femoris, **(B)** Biceps Femoris, **(C)** Tibialis Anterior, and **(D)** Gastrocnemius. In each subplot, the upper panel displays the ensemble mean (solid and dashed lines) and standard deviation (shaded bands) trajectories of muscle activity. The lower panel illustrates the corresponding SPM{t} statistic trajectory (solid black line). The horizontal dashed grey lines indicate the critical threshold calculated for a significance level of alpha = 0.05. Grey shaded vertical regions denote suprathreshold clusters where the t-statistic exceeds the critical threshold, indicating a statistically significant difference between limbs (p < 0.05). The horizontal axis represents the normalized movement cycle (0–100%).

**Figure 1** presents the normalized sEMG RMS mean curves and standard deviations of four major lower limb muscles for both legs across the entire single-leg CMJ cycle (0–100%). SPM analysis revealed significant differences between limbs in the RF (**Figure 1A**) at 56–58%, 69%, and 82% of the CMJ cycle (p < 0.05; peak d = 1.21, 0.64, and 0.70, respectively), with higher activation observed in the left leg. For the BF (**Figure 1B**), significant differences were found at 4%, 45–47%, and 83–88% of the cycle (p < 0.05; peak d = 0.80, 0.92, and 1.04, respectively), with the left leg again showing greater activation. The TA (**Figure 1C**) showed significant differences during the early phase of the cycle (1–7%), with higher activation in the left leg (p < 0.05; peak d = 1.17). For the MG (**Figure 1D**), no statistically significant differences were detected throughout the CMJ cycle (p > 0.05). Outside the identified time windows of significant difference, the bilateral muscle activation curves were nearly identical, showing no significant asymmetry. Although the overall muscle activation patterns were largely symmetrical between limbs, localized asymmetries in activation were evident during the early and mid-phases of the vertical jump.

**Figure 2:**
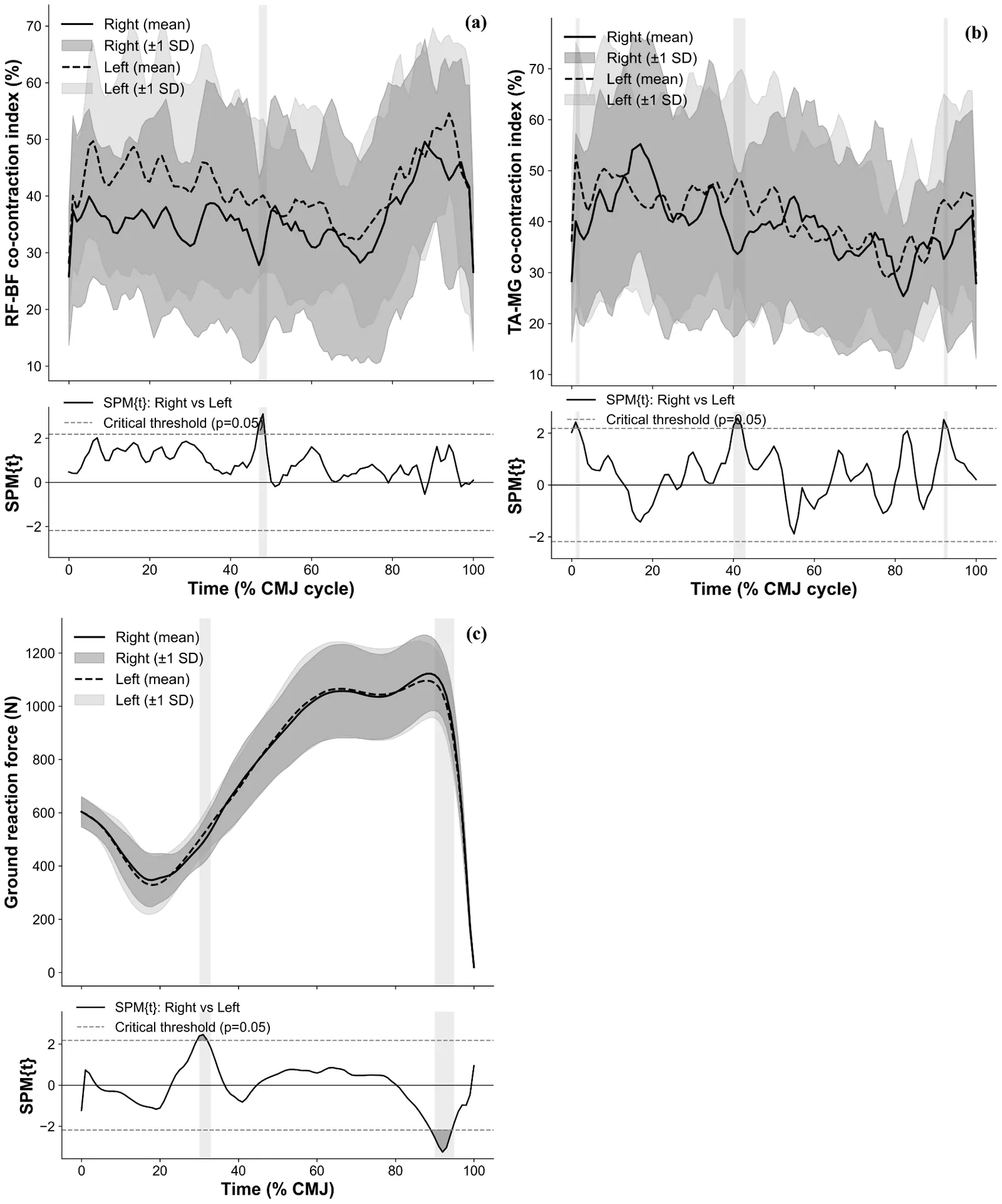
Statistical parametric mapping (SPM) analysis of lower limb muscle co-activation indices (CCI) and vertical ground reaction force (vGRF) during the single-leg countermovement jump (CMJ). Panels display the CCI for **(A)** the Rectus Femoris and Biceps Femoris (RF-BF) pair, **(B)** the Tibialis Anterior and Medial Gastrocnemius (TA-MG) pair, and **(C)** the vGRF. In each subplot, the upper panel presents the ensemble mean (solid and dashed lines) and standard deviation (shaded bands) trajectories. The lower panel illustrates the corresponding SPM{t} statistic trajectory (solid black line). The horizontal dashed grey lines indicate the critical threshold calculated for a significance level of alpha = 0.05. Grey shaded vertical regions denote suprathreshold clusters where the t-statistic exceeds the critical threshold, indicating statistically significant bilateral differences (p < 0.05); these clusters mark the movement phases in which the differences occurred—those in the early cycle correspond to the braking phase, those around 30–48% to the braking-to-propulsion (rapid force-development) transition, and those around 90–94% to the late propulsion phase preceding take-off. All data are time-normalized to the movement cycle (0–100%).

**Figure 2** illustrates the time-series changes of lower limb muscle group CCI and vGRF for both legs during the single-leg CMJ, along with the corresponding SPM analysis results. In CCI_RF-BF_ (**Figure 2A**), a significant difference was observed at 47–48% of the CMJ cycle (p < 0.05; peak d = 1.00), with the left leg showing significantly higher CCI_RF-BF_ than the right. For CCI_TA-MG_ (**Figure 2B**), significant differences were detected at 1%, 40–42%, and 92% of the cycle (p < 0.05; peak d = 0.63, 1.04, and 0.68, respectively), also with greater values in the left leg. Regarding vGRF (**Figure 2C**), the vGRF was significantly higher in the left leg during 30–32% of the cycle (p < 0.05; peak d = 0.68), while the right leg showed significantly higher vGRF during 90–94% (p < 0.05; peak d = 0.90). Outside these specific time windows, none of the variables exceeded the significance threshold (p > 0.05), indicating that the overall movement pattern was relatively symmetrical between limbs, with only localized differences during the rapid force production phase and late peak stage.

**Table 1** presents the means and standard deviations of CCI_RF-BF_, CCI_TA-MG_, vGRF, and their corresponding asymmetry indices. Descriptive analysis showed relatively high asymmetry indices for CCI_RF-BF_ and CCI_TA-MG_, with higher CCI_RF-BF_ on the left side and higher CCI_TA-MG_ on the right. In contrast, the asymmetry index for vGRF was very low, indicating a high degree of symmetry. Correlation analysis results (see **Table 2**) revealed a significant positive correlation between climbing time and ASI_vGRF_ (r = 0.61, p = 0.028), suggesting that greater vGRF asymmetry was associated with longer climbing times. However, no significant correlations were found between climbing time and either ASI_RF-BF_ (r = -0.14, p = 0.641) or ASI_TA-MG_ (r = -0.25, p = 0.418).

**Table 1:** Means and standard deviations of muscle co-activation index (CCI), vertical ground reaction force (vGRF), and asymmetry index (ASI) during the single-leg countermovement jump task (n = 14).

Variable	Left	Right	ASI (%)
CCI_RF-BF_	41.76 ± 5.7	36.42 ± 11.37	28.7 ± 25.73
CCI_TA-MG_	39.48 ± 13.02	41.6 ± 13.09	29.22 ± 27.5
vGRF (N)	755.52 ± 78.69	756.42 ± 77.79	1.26 ± 0.62

RF, Rectus Femoris; BF, Biceps Femoris; TA, Tibialis Anterior; MG, Medial Gastrocnemius.

**Table 2:** Descriptive statistics of climbing time and its correlations (r) with asymmetry indices (ASI) of vertical ground reaction force (vGRF) and muscle co-activation (n = 14).

Variable	Mean ± SD	r (ASI_vGRF_)	r (ASI_RF-BF_)	r (ASI_TA-MG_)
Climbing time	8.47 ± 2.01	0.61*	-0.14	-0.25

RF, Rectus Femoris; BF, Biceps Femoris; TA, Tibialis Anterior; MG, Medial Gastrocnemius. *Indicates a significant correlation (p < 0.05).

## 4 Discussion

Although the unilateral CMJ cannot fully replicate the whole-body coordination of speed climbing, it serves as a valuable isolated model to examine intrinsic neuromuscular control strategies of the lower limbs. Within this framework, this study aimed to examine differences in neuromuscular control strategies and force output between limbs during single-leg jumping, and to further analyze the extent to which force output asymmetry and muscle co-contraction asymmetry are associated with speed climbing performance, as measured by climbing time. The results indicated that during both the braking phase (involving TA and CCI_TA-MG_) and the propulsion phase (involving RF, BF, and CCI_RF-BF_), the left limb showed significantly higher levels compared to the right. This suggests that the left leg may have played a dominant role in movement control through greater neural input and joint stiffness. This high-stiffness control characteristic may have enabled the left leg to lead during the rapid force development phase, whereas the right leg demonstrated a compensatory advantage during the late peak phase of the jump. Such temporal complementarity likely contributed to maintaining overall symmetry in vGRF. Although considerable asymmetry was observed in knee and ankle joint co-contraction, correlation analysis indicated that the balance of external kinetic output was the variable most closely associated with climbing performance—climbing time was significantly positively correlated with vGRF asymmetry. This suggests that, when compensatory strategies maintain symmetry in bilateral push-off forces, asymmetries at the muscular control level may not be associated with impaired performance.

Neuromuscular asymmetries between lower limbs may not only increase the risk of sports injuries but have also been closely associated with reduced athletic performance (Bishop et al., 2018; Helme et al., 2021). Therefore, identifying asymmetry characteristics in speed climbers’ lower limbs is of great importance for developing evidence-based training and injury prevention strategies. The present study found significantly higher muscle activation levels in the left lower limb across several key muscles, specifically in the TA during the braking phase, and in the RF and BF during the propulsion and peak maintenance phases. These results differ to some extent from the findings of Zhang et al. (2025), who reported no significant differences in the integrated sEMG values of RF and MG between limbs during CMJ. However, they did report a high asymmetry index of 22.03% for RF. This discrepancy may result from methodological differences in data processing. Zhang et al. (2025) used a traditional approach that integrated the entire movement cycle, which may have masked significant differences occurring within short, specific phases. In contrast, the present study employed Statistical Parametric Mapping, which allows for dynamic analysis of sEMG signal trajectories across the entire jump cycle and is therefore more sensitive to detecting activation differences limited to specific time windows. Our observation of higher left-TA activation during the braking phase is broadly consistent with the left-lateralized neuromuscular pattern reported by Guo et al. (2019), who found greater fatigue-related decreases in sEMG median frequency in the left than the right limbs during a 15-m speed climb. It should be noted, however, that their left-lateralized pattern was most pronounced in the upper-limb muscles, and that lower-limb sEMG amplitudes did not differ significantly in their study. Our finding therefore represents a novel observation rather than a confirmation of theirs, and raises the possibility that a left-lateralized control tendency may extend to the lower limbs and transfer to non-sport-specific tasks—a hypothesis that warrants further investigation.

To our knowledge, no previous study has systematically investigated asymmetries in knee and ankle joint co-contraction in climbers. This study is the first to reveal that speed climbers exhibit generally high levels of asymmetry in knee and ankle CCI. Further SPM analysis identified specific temporal characteristics of this asymmetry: the left knee and ankle showed significantly higher CCI during the propulsion phase, and the left ankle maintained elevated CCI during the peak torque phase. These findings suggest that in the complex kinetic environment of high-speed vertical climbing, the two lower limbs adopt different functional roles and distinct neuromuscular control strategies. This is in line with the classical “functional asymmetry theory” described in gait and sports biomechanics. Sadeghi et al. (2000) noted that the dominant limb (typically the right) primarily generates propulsion, while the non-dominant limb (typically the left) contributes more to support and postural stability. It should be noted, however, that Sadeghi et al. (2000) examined limb dominance during gait; its application to the single-leg CMJ and to speed climbing is therefore drawn as a theoretical analogy rather than as direct evidence, and warrants confirmation in sport-specific contexts. The higher CCI observed in the left limb may reflect a neuromuscular strategy to compensate for lower control precision on the non-dominant side, achieved through increased joint stiffness. Baratta et al. (1988) reported that greater co-contraction of antagonistic muscles can enhance dynamic joint stability, improving resistance to external perturbations and shear forces. During peak force production in speed climbing, high vertical ground reaction forces demand joint stability to minimize energy loss. One possible interpretation is that the higher CCI on the left side reflects a strategy of increasing joint stiffness to create a more rigid support point, thereby enhancing force-transmission efficiency and reducing instability risk, whereas the lower CCI on the right may facilitate explosive extension with reduced internal resistance and greater movement velocity. This account is consistent with Cormie et al. (2011), who suggested that reducing co-contraction is key to improving neuromuscular power output by minimizing opposing joint torques. Given that the present participants were elite climbers with national-level competitive achievements, this left-lateralized co-contraction pattern is more likely to reflect a refined, training-induced stabilization strategy than an uncorrected, inefficient compensation; nevertheless, the possibility of a compensatory component cannot be entirely excluded. It should be noted, however, that these interpretations are derived from an isolated single-leg CMJ task and require further verification before being generalized to whole-body speed climbing. Overall, the elevated CCI in the left knee and ankle joints may represent a functional neuromuscular adaptation that could enhance non-dominant limb stability during high-speed movement, possibly at the cost of some metabolic efficiency.

Moreover, this study found that despite considerable neuromuscular asymmetry in co-contraction at the knee and ankle joints, bilateral vGRF demonstrated a relatively symmetrical overall distribution. Building on the perspective of Sadeghi et al. (2000), we interpret this finding as suggesting that local asymmetries do not necessarily result in global behavioral abnormalities. Further temporal analysis indicated that this apparent symmetry is actually a functional compensation strategy, achieved through differential force output at different phases of the movement. Specifically, the left leg produced greater output during the rapid force development phase, while the right leg showed stronger output during the late peak phase of the jump. This mechanism suggests that the limbs collaborate in a temporally complementary manner, with each side contributing distinct functional roles such as rapid initiation and late-phase maintenance, thereby maintaining global kinetic stability. This observation not only reveals the dynamic mechanism underlying vGRF symmetry but also supports and extends the findings of Zhang et al. (2025), who reported in bilateral CMJ tests that the left leg exhibited superior rate of force development while the right produced greater peak force, although no statistically significant differences were found. The significant results observed in the present study may stem from differences in analytical methods. As Sadeghi et al. (2000) noted, averaging or aggregating data across the entire cycle may “smooth out” real fluctuations occurring in specific time windows, thereby obscuring significant local differences. In contrast, the SPM technique used in this study enables point-by-point analysis of time-series data during the movement process, offering advantages in detecting localized dynamic differences and capturing important temporal features that may be overlooked in conventional analyses. In terms of correlation analysis, this study found no significant association between asymmetry in knee and ankle CCI and climbing performance. Given the small sample size and limited statistical power, this null result should be interpreted with caution and does not necessarily indicate that neuromuscular asymmetry is irrelevant to performance; it warrants re-examination in larger samples. However, the asymmetry index of force output was significantly positively correlated with climbing time—that is, greater asymmetry was associated with longer completion times and poorer performance. This result aligns with the findings of Zhang et al. (2025), who reported that rate of force development and peak force were closely associated with speed climbing performance. The main distinction of the present study lies in its systematic evaluation of bilateral asymmetry from the perspective of global mechanical output, further supporting the view that asymmetry appears to be associated with poorer sport-specific performance mainly when local compensatory mechanisms fail to maintain global kinetic balance.

Although this study provides new insights into bilateral differences in speed climbers, several limitations should be considered when interpreting the results. First, the sample size was relatively small (n = 14), reflecting the limited availability of elite, national-level speed climbers, who constitute a small and difficult-to-recruit population. Although the primary correlation represented a large effect (r = 0.61), a *post-hoc* power analysis (G*Power, bivariate normal model) indicated an achieved power of approximately 0.793 for this correlation, approaching but not reaching the conventional 0.80 criterion. The present findings should therefore be interpreted with caution, and confirmation in larger cohorts is warranted. Second, the cross-sectional design limits causal inference. While a significant correlation between force output asymmetry and climbing performance was observed, the direction of causality remains unclear. Future longitudinal intervention studies are needed to clarify this relationship. Third, knee-flexion angle during the countermovement was not standardized but self-selected; although this was a deliberate choice intended to preserve each athlete’s natural movement strategy and individual lower-limb stiffness, it may introduce some inter-individual variability in countermovement depth, and future studies may consider recording or controlling knee angle to further clarify its effect. Relatedly, limb dominance and habitual starting-leg strategy were not recorded; the observed left–right differences are therefore interpreted as functional, task-related differentiation rather than as necessarily reflecting limb dominance, and future studies should collect these variables. Lastly, the study sample consisted exclusively of male athletes. Considering the known sex-specific differences in Q-angle, muscle distribution, and hormonal profiles, patterns of lower limb asymmetry may differ in female athletes. Therefore, the conclusions drawn from this study are limited to high-level male climbers. Future research should include female athletes to establish a more complete biomechanical foundation for speed climbing.

## 5 Conclusion

Through temporal-phase analysis, this study revealed functional lateral differences in the lower limbs of speed climbers and their association with performance. The results showed that during the single-leg countermovement jump task, athletes exhibited clear functional differentiation between limbs. The left limb demonstrated higher muscle activation (TA, RF, BF) and joint co-contraction levels during key phases such as braking, propulsion, and peak maintenance, suggesting a possible role in support and postural stabilization. In contrast, the right limb appeared to be more involved in explosive propulsion. Despite the evident asymmetry in neuromuscular control, the overall vGRF remained relatively symmetrical, supporting the notion that local asymmetry does not necessarily lead to global behavioral abnormalities. However, the significant positive correlation between vGRF asymmetry and climbing time suggests that greater symmetry in external force output may be associated with better sport-specific performance. Based on these findings, training practice may address both functional differentiation and overall kinetic symmetry. For the left limb, training may emphasize isometric contractions and exercises on unstable surfaces to enhance joint stiffness and stability, whereas for the right limb, plyometric training may be prioritized to improve explosive strength. In addition, bilateral synchronous strength training may help maintain coordinated force production and preserve balance in push-off output between limbs, thereby supporting speed-climbing performance.

## Data Availability

The raw data supporting the conclusions of this article will be made available by the authors, without undue reservation.
